# Editorial: New insights in nanotechnology for plant stress management

**DOI:** 10.3389/fpls.2023.1319936

**Published:** 2023-11-09

**Authors:** Besma Sghaier-Hammami, Sofiene B.M. Hammami

**Affiliations:** ^1^ Laboratory of Bioaggressors and Integrated Protection in Agriculture LR14AGR02, The National Agronomic Institute of Tunisia, University of Carthage, Tunis, Tunisia; ^2^ Institut National Agronomique de Tunisie, Laboratoire LR13AGR01, Université de Carthage, Tunis, Tunisia

**Keywords:** nanotechnology, plant, agriculture, sustainability, biotic and abiotic stress

Agriculture on our planet faces many challenges due to environmental stresses (biotic and abiotic), limiting plant growth and causing yield loss, thus inciting food crises across the world (Fisher et al., 2018). To attain sustainable agricultural production and ensure food safety, there is a demand to elevate the stress forbearance in plants. In recent years, nanotechnology has emerged as a viable platform in the era of agriculture, attracting the interest of researchers from a variety of disciplines, for resolving challenges associated with biotic and abiotic stresses to assure agricultural sustainability (Duhan et al., 2017; Du et al., 2020; Hossain et al, 2020; Baazaoui et al, 2021; Baazaoui and Sghaier-Hammami, 2021; Baazaoui et al., 2023). Nanoparticles (NPs) have positive impacts on plant growth and development and tolerance against biotic and abiotic stresses, by increasing the activities of antioxidants (Emamverdian et al., 2020; Ahmed et al., 2021), and maintaining the efficiency of the photosynthetic apparatus (Soares et al., 2018). Nanotechnology application in agriculture will help to reduce the environmental release of agrochemicals like pesticides, herbicides, and fertilizers (Adisa et al., 2019; Ali et al., 2021). This will not only help in reducing pollution but also help in reducing the input cost of cropping. Five articles focused on providing comprehensive reviews of the role of NPs utilized to tolerate different abiotic stresses in plants.

The work on the nickel-silica nanoparticles by Abdallah et al. provides useful insights into the significance of these NPs on the suppression of bacterial (Xoo) leaf blight (BLB) disease in rice (*Oryza sativa* L.). The obtained results demonstrate that the saffron (*Crocus sativus* L.) stigma extracts represent a good candidate to synthesize pure nanocomposite (Ni-SiO_2_). The *in vitro* experiments showed that Ni-SiO_2_ composite inhibits bacterial growth in nutrient broth and hindered biofilm development of Xoo cells in a dose-dependent manner. The research showed that the incubation of Xoo with Ni-SiO_2_ NPs composite (200 μg/ml) drastically increased the apoptosis of the bacterial cells to 99.61%. The application of Ni-SiO_2_ NPs composite at a concentration of 200 mg/ml significantly improves plant growth (leaf length and high, shoot and root length, plant fresh and dry weight) and reduces disease severity of BLB in rice.

In the review by Rasheed et al. these authors looked at the use of different metal-based NPs to protect plants against drought stress (DS). The authors resume the different modes of NPs synthesis such the conventional methods (chemical and physical processes) and green synthesis which have an excellent photo-catalytic activity and provides an excellent environmentally friendly option. The study by Rasheed et al. showed that NPs uptake in plants can occur through leaf cuticles, stomata, trichrome, hydathodes, and cell cytoplasm, wounded regions, and root junction. In more, this review provides an overview of the biological functioning of NPs which depends on their concentrations, properties, and methods of application. The role of NPs in drought condition and their beneficial effect on plants has been demonstrated. The NPs have the ability to protect crop plants against DS by enhancing nutrient and water uptake, increase plant growth, improve photosynthetic efficiency, accumulation of osmolytes hormones, and phenolics, antioxidant activities, and gene expression, thus providing better resistance to plants DS.

In the review by Halawani et al. the authors provided an overview of the implementation of Carbon nanoparticles (CNPs) in salinity stress tolerance. By activating plant defense systems via the stimulation of proline metabolism, anthocyanin metabolism, and polyamine metabolism, CNPs can minimize the deleterious impacts of salinity stress in radish sprouts. In more, seed nano-priming boosted the antioxidant capacity by increasing antioxidant metabolites such as (polyphenols, flavonoids, polyamines, anthocyanin, and proline).

The high potential of ZnO nanoparticles as a nano-fungicide against *Magnaporthe grisea* was described by (Ghamari et al.). The response of fungi to ZnO NPs was evaluated using RNA sequencing (RNA-seq). The authors have observed that ZnO NPs primarily act on the fungal cell membrane and then induce oxidative stress through the production and accumulation of reactive oxygen species. As consequence, the fungal catalytic system is disrupted, resulting into the inhibition of ROS scavenging. Therefore, ROS accumulated inside the cell, the intracellular homeostasis is disrupted, oxidative stress occurred, which damaged macromolecules, eventually leading to fungal cell death.

Among the contributions made to this topic, the original article written by Ayub et al. elucidated the negative impact of NPs in crops, by using cerium oxide nanoparticles (CeO_2_). CeO_2_ NPs were toxic for corn growth by altering root barrier formation which could potentially affect plant root anatomy. When applied in combination with Cd, no significant toxicity on corn growth was observed as the 1,000 mg kg^-1^ dose increased shoot growth. The researchers showed also that the effect on root growth was variable due to the divergent uptake of nutrients, beneficial elements, Ce, and Cd.

## Concluding remarks and future perspectives

In summary, the work presented in this Research Topic showed how different types of NPs could be effectively applied to mitigate biotic and abiotic stress in different plants, by improving crop productivity and enhancing photosynthetic machinery and improving antioxidant system. However, for the safe and ethical application of nanotechnology in agriculture, thorough risk assessment and ethical considerations are essential, since NPs could have a toxic effect on plants, leading to their death.

## Author contributions

BS-H: Writing – original draft, Writing – review & editing. SBMH: Writing – review & editing.
